# Target Organ Damage and the Long Term Effect of Nonadherence to Clinical Practice Guidelines in Patients with Hypertension: A Retrospective Cohort Study

**DOI:** 10.1155/2017/2637051

**Published:** 2017-06-13

**Authors:** Tadesse Melaku Abegaz, Yonas Getaye Tefera, Tamrat Befekadu Abebe

**Affiliations:** College of Medicine and Health Sciences, School of Pharmacy, Department of Clinical Pharmacy, University of Gondar, Gondar, Ethiopia

## Abstract

**Background:**

There was limited published data on target organ damage (TOD) and the effect of nonadherence to practice guidelines in Ethiopia. This study determined TOD and the long term effect of nonadherence to clinical guidelines on hypertensive patients.

**Methods:**

An open level retrospective cohort study has been employed at cardiac clinic of Gondar university hospital for a mean follow-up period of 78 months. Multivariate Cox regression was conducted to test associating factors of TOD.

**Results:**

Of the total number of 612 patients examined, the overall prevalence of hypertensive TOD was 40.3%. The presence of comorbidities, COR = 1.073 [1.01–1.437], AOR = 1.196 [1.174–1.637], and nonadherence to clinical practice guidelines, COR = 1.537 [1.167–2.024], AOR = 1.636 [1.189–2.251], were found to be predicting factors for TOD. According to Kaplan-Meier analysis patients who were initiated on appropriate medication tended to develop TOD very late: Log Rank [11.975 (*p* = 0.01)].

**Conclusion:**

More than forty percent of patients acquired TOD which is more significant. Presence of comorbidities and nonadherence to practice guidelines were correlated with the incidence of TOD. Appropriate management of hypertension and modification of triggering factors are essential to prevent complications.

## 1. Background

Hypertension (HTN) is deemed the major cause of morbidity and mortality worldwide. It affects approximately 1 billion individuals globally [1]. HTN ranked as the leading single factor for Global Burden of Diseases, most notably in Sub-Saharan Africa and South Asia regions where a substantial proportion of the world's population resides. In Sub-Saharan Africa, prevalence of raised blood pressure was around 30% [2]. A recent systematic review showed that the prevalence of hypertension among Ethiopian population was estimated to be 19.6%. Its burden is supposed to be increasing due to the growth and aging of the population around the world [3, 4]. Death from hypertension arises from target organ damage (TOD) such as cardiovascular, cerebrovascular, and renovascular accidents. TOD is the structural and functional impairment of major body organs due to elevated blood pressure (BP). These vital organ impairments including left ventricular hypertrophy (LVH), proteinuria, retinopathy, and vascular dementia are collectively referred as target organ damage [5]. The heart, brain, and kidney are the main target of elevated blood pressure because these organs take the large share of the blood circulating in the vasculature [6]. The cardiovascular complications include left ventricular hypertrophy (LVH), heart failure (HF), ischemic heart disease (IHD), and cardiac arrhythmias. Cerebrovascular injury resulted in the form of ischemic and hemorrhagic stroke events, whereas the renal effect ranges from asymptomatic structural damage to end stage renal disease with massive protein urea. Hypertensive patient also occasionally presented with retinopathy, peripheral vascular disease, and sexual dysfunction [7].

Globally, about 62% of cerebrovascular diseases and 49% of ischemic heart disease are attributable to elevated blood pressure (BP) [8]. In developing countries, its morbidity and mortality are increasing due to a change in life style. Patients with uncontrolled blood pressure (UBP) are the most at risk for TOD. A cohort study has reported that poorly controlled hypertension is independently associated with mortality, cardiovascular risk, and disease progression [9]. Some other factors such as advanced age (longevity), overweight, and obesity were also found to be related to the incidence of cardiovascular complications in hypertensive patients [10].

The burden of TOD could be modified by early diagnosis, treatment, and close follow-up of HTN. Periodic review of treatment modalities has been tried globally so as to bring the best treatment approach [11]. New diagnostic maneuvers and treatments are being introduced every year to get blood pressure lower. Other interventions like combination therapy are frequently practiced to achieve optimal control of BP [12]. These measures achieve effective reduction of TOD through blood pressure (BP) control [13]. Despite these efforts, the incidence TOD is dramatically increasing worldwide due, partially, to the lack of implementation of clinical practice guidelines (CPGs). Estimation of the magnitude of TOD among HTN patients and investigation of potential risk factors for TOD will enable us to reduce or modify the burden of TOD [14]. The current study has been expected to address epidemiologic nihilism of TOD. To the researchers' knowledge, there was limited published data on the determinants of TOD in developing countries including Ethiopia. This study focused on the prevalence and predicting factors of TOD on hypertensive patients attending tertiary care hospital, north Ethiopia, during the year 2015-2016.

## 2. Methodology

### 2.1. Study Area and Study Population

Gondar university hospital (GUH) is a teaching referral hospital that serves 5 million people in northwest Ethiopia. It has both in-patient and outpatient departments (OPDs). The medical OPD comprised the newly established 1000-bed medical ward including chronic diseases. Chronic disease follow-up is given at this hospital for hypertensive, diabetic, asthmatic, and heart failure patients from the area. The follow-up period is arranged between weeks and months based on the choice of the patient and appointment for further investigations by senior physicians. All hypertensive patients that do have follow-up at GUH were our source population. Patients who had complete documentation were included under the study population. All age groups were considered for the study.

### 2.2. Study Design and Study Period

An open level retrospective cohort study has been applied by which all eligible patients are enrolled in the study as soon as they are registered to cardiac clinic till the completion of the study. Patients were retrospectively followed for a mean period of 78 months. During this time patients might develop TOD, be lost to follow-up, or remain without complications. TOD at the time of presentation was also included. Patients on treatment were particularly evaluated for appropriateness of their therapy versus the incidence of TOD.

### 2.3. Sample Size Determination and Sampling Technique

**Figure 1:**
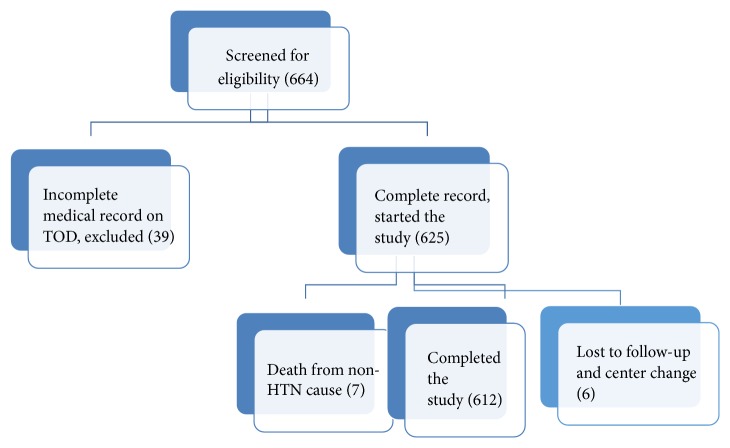
The inclusion of subjects in the cohort study.

About 664 hypertensive patients that return to clinic at their appointment dates were evaluated for eligibility.  Figure 1 illustrated the stepwise enrollment of study subjects.

### 2.4. Inclusion and Exclusion Criteria

Patients who have had a complete medical record and who have a routine and regular follow-up were included in our study. Patients who were lost to follow-up and death from nonhypertension cause were excluded.

### 2.5. Variables

The types of complications were our dependent variables while date of appointment, BP level, concordance with the national treatment guidelines and the eighth joint national conference (JNC-8), regimen change, age, sex, and type of therapy were the independent variables.

### 2.6. Data Entry and Analysis

Data was entered to and analyzed by SPSS software for windows version 21. A person-time method was taken to calculate the incidence rate of TOD. Cox regression was conducted to test associating factors of target organ damage. Kaplan analysis was done to evaluate the effect of inappropriate drug selections on TOD. *p* value was set at 0.05 with 95% confidence interval. Incidence rate (IR) of each TOD was determined by taking into account the actual observation time of each subject during the study period. It was calculated based on the following formula:

(1) IR=number  of  new  cases  addedd  each  yeartotal  person−time  of  observtion  in  poplaton  at  risk,IR=number  of  new  hypertensive  cases  addedd  each  yeartotal  person−time  of  observation  in  hypertensive  patients.

### 2.7. Data Collection Procedure

Data was collected by trained data collectors who have been working at University of Gondar, School of Pharmacy. A structured questionnaire containing sociodemographic data, type of complication, year of complication, and follow-up period was designed by the investigators. Data collectors directly fill the questionnaire by searching the relevant information available on patients' medical card. Echocardiography and electrocardiography results were referred during our data collection. Blood pressure measurement took place during the last follow-up. Each subject underwent further physical examination to determine clinical features of LVH, HF, stroke, renal failure, and fundoscopy for hypertensive retinopathy. Hypertensive cardiac damage was defined by the presence of electrocardiographic LVH based on the voltage criteria [15]. Abnormal heart rhythms, wall motions, and tension were taken from echocardiographic results using M-mode and two-dimensional (2D) echocardiography. Renal damage was confirmed based on the presence of microalbuminuria as determined by spot urine albumin-to creatinine ratio. CT scan results were investigated to distinguish stroke events [13]. Erectile dysfunction was evaluated with the International Index for Erectile Function questionnaire.

*Ethical Considerations*. Permission from School of Pharmacy was obtained in the form of written consent. Ethical clearance was requested and obtained from clinical director of University of Gondar hospital.

## 3. Results

### 3.1. Sociodemographic and Clinical Characteristics of Respondents

Of the total 664 patients, 612 who fulfilled the inclusion criteria were enrolled for the study which gives a response rate of 92.2%. The mean age of the participants was 52.43 ± 13.2. The mean follow-up period was 78 months. About 341 (55.72%) study subjects were females. Majority of the participants were having both systolic and diastolic hypertension (70.7%). Nearly forty-five percent (44.94%) of the respondents were appointed to rerun to clinic for the next visit every two months. About forty percent of the study participants encountered TOD (40.35) Table 1.

**Figure 2:**
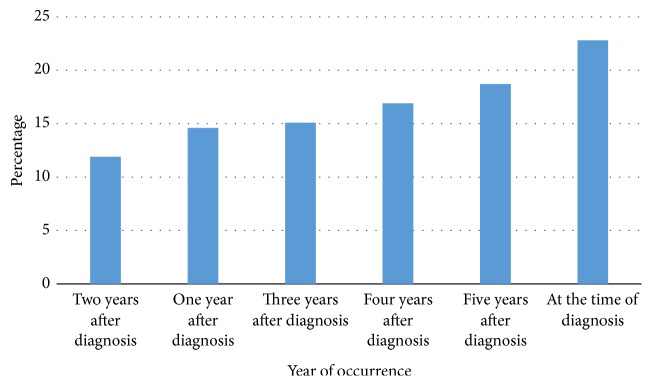
The occurrence of hypertensive complications each year.

Most of the complications found to be appeared at the time of presentation (22.8%). Complications declined 1 to 3 years after diagnosis (11.9%). But, after five years of diagnosis the complication became more common again (18.7%) (Figure 2).

### 3.2. Incidence Rate of Hypertensive TOD

The incidence rate of HF was around 30.8 cases/1000/person per year (PY). The second most common TOD is IHD (22 cases/1000/PY). Significant number of patients also experienced hypertensive urgencies (17.55/1000/PY). Sexual dysfunction is the least reported organ damage (0.49/1000/PY) each year (Table 2).

The most prevalent TOD was HF (11.6) followed by ischemic heart disease (8.3). The least common TOD was sexual dysfunction (0.67), retinopathy (0.9), and AF (0.9) (Table 3).

A Cox regression result indicated that several factors were associated with the occurrence of TOD in hypertensive patients. Physicians nonadherence to clinical practice guidelines (CPGs) was associated with the occurrence of TOD, COR = 1.537 [1.167–2.024], AOR = 1.636 [1.189–2.251]. Absence of regimen change, COR = 1.702 [1.240–2.336], AOR = 1.857 [1.325–2.602], and the presence of comorbidities, COR = 1.073 [1.01–1.437], AOR = 1.196 [1.174–1.637], increased the risk of TOD. In addition, patients with seven-year duration of hypertension were nearly three times more likely to develop complications, COR: 2.951 [2.651–5.535], AOR: 2.974 [2.631–7.072]. Moreover, monotherapy with beta-blockers was found to increase the incidence of TOD COR: 2.951 [2.651–5.535] and AOR: 2.974 [2.631–7.072]. However, sex, age, type of therapy, and residence were not significantly associated with the rate of TOD (Table 4).

**Figure 3:**
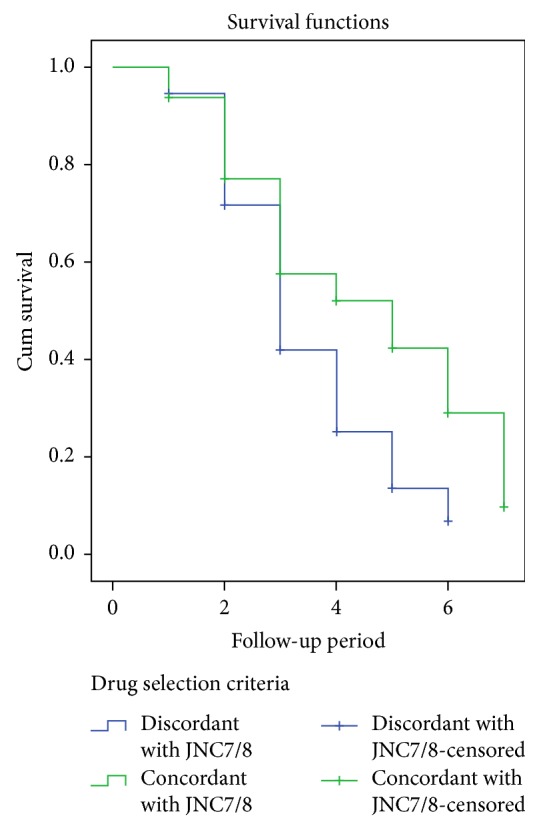
Kaplan Meier analysis curve of TOD based on the concordance of prescribed medications: a 78-month follow-up.

When patients were followed for a period of 78 months in terms of treatment concordant with guidelines, there was significant difference in the rate of TOD among patients: Log Rank 11.975 (*p* = 0.01). Concordant patients develop TOD very late as compared to those who receive medication without the standard treatment guideline. The mean progression free period for discordant patients was shorter than concordant patients (Figure 3).

## 4. Discussion

Hypertensive complications manifested as end organ damage are the major indicator of advanced disease. The timing of appearance of organ damage was dependent on the presence of triggering factors. This study aimed to evaluate the burden and determinants of TOD in hypertensive patients attending GUH. It was found that the overall cases of TOD were 40.3%. Comparative result (43.1%) was reported in Nigerian hypertensive patients [7]. Another study revealed 44.5% TOD in hypertension patients attending primary care [16]. In the present study most of the complications were found to appear at the time of presentation (22.8%). This might be due to the late presentation of patients and underdiagnosis of cases [17–19]. Complications declined 1 to 3 years after diagnosis due to the initiation of therapy and life style modifications [20]. But after five years the complication became common again; this might be due to the fact that the therapy is overcome due to the progression of the disease in resistance to HTN and aging of the patients [21, 22].

The cumulative incidence of HF was 30.8% which was relatively higher rate as compared to a finding reported by Sahle et al. 6.26/1000/year [23]. The high incidence of HF in our study might be due to the lack of adherence to CPGs in order to prescribe medications with compelling indication which could have reduced the progression of structural heart disease into overt HF [24]. Moreover the regression of LVH might be delayed if appropriate medicine is not initiated because antihypertensive drugs have different effect on ventricular regression [25]. In addition the high rate of IHD could contribute to the presence of HF as IHD might gradually progress to HF [26]. The prevalence of LVH was 2.2% in the present study. High magnitude of LVH was reported in Spain (22.9%), Nigeria (27.9%), and Greece (33%) [7, 16, 27]. The lower finding of LVH in our study could be attributed to the rapid progression of LVH into HF which diminishes the number of LVH cases [28]. The rate of hypertensive retinopathy was below one percent in the present study and it was nearly 2% in Nigerian patients [6]. On the contrary, it was significantly higher (39.9%) in Iranian hypertensive patients (HPs); this might be due to the high level of sever BP pressure, in Iranian population which might directly impose with the occurrence of complications. Moreover, there was a significant difference in the study design and period between the two studies [29].

In this study, nearly 5.5% of hypertensive patients encountered stroke events. Almost similar result (6.3%) was observed in multicenter study from Greece [16]. Choi and Park reported a relatively higher rate of stroke in Korean HPs. This might be due to the longer follow-up period and nearly one-half of the respondents were having AF which is the major risk factor for stroke in Korean HPs [30]. Our studies revealed nearly one percent prevalence of AF. A nationwide study from Thailand stated that prevalence of AF in hypertensive individuals was found to be (3.46%) [31]. The prevalence of atrial fibrillation was 16.4% in Nigerian population. This disparity could be attributed to low rate of BP control and comorbid peripheral vascular disease in Nigerian HPs [7].

It was found that multiple factors were found to be implicated with the incidence of TOD to long standing hypertensive patients. The frequency of regimen changes independently affects the occurrence of TOD. Periodic regimen changes were associated with low incidence of TOD. This might be due to the initiation of a single therapeutic agent at subtherapeutic dose [11]. Hence, the regimen change might allow new and appropriate therapeutic options including combination therapies [32]. Sex was not correlated with higher incidence of TOD. However, Papazafiropoulou et al. has reported that TOD was more prevalent in males than in females [16]. Other studies identified that male gender was associated with the incidence of stroke [33] and AF [20]. A large survey from Chinese HTN population has discovered that older age, male gender, and SBP were independently associated with cardiovascular, coronary, and renal complications [34]. However, in this study any age group and type of HTN was not related with cardiovascular or cerebrovascular damage.

The present study has also found that the coincidence of comorbidities increased the risk of TOD nearly 1.5 times. This is due to the fact that these special populations predisposed for other noncommunicable disease states which might independently cause TOD [35]. Moreover, when patients acquire more than one disease state at the same time, they are forced to take multiple medications which is commonly referred polypharmacy. The effect of polypharmacy is escaping of one or more doses of a certain drug due to pill burden and suffering from side effects which leads to uncontrolled HTN. Previous studies indicated that polypharmacy was independently correlated with uncontrolled HTN which in turn determines the incidence of TOD [35–37]. Medication recommendations to the national standards guidelines were found to substantially reduce the number of TOD. It is true that the provision of medications as per national standard treatment guidelines enables adequate control of blood pressure and regression of TOD through the administration of the right therapeutic agent for the right patient [38]. This 78-month follow-up of subjects indicated that patients that took appropriate medications develop TOD very late as compared to those who receive medication without the standard treatment guideline. The mean progression free period for discordant patients was shorter than concordant patients. A study compared treatment guidelines and BP control also stated that patients who were prescribed an antihypertensive regimen that is JNC concordant were having optimal control of BP [39]. The basic aim of adherence to standard treatment guidelines in the treatment of hypertension is to achieve adequate disease control, since controlled hypertension results delayed complications and progression of target organ damage.

Overall, this study has provided a baseline evidence on the burden and determinants of TOD in Ethiopia. However, it did not investigate the impact of obesity, metabolic syndrome, and social habits such as smoking history on TOD. It is also carried out in a single center with limited number of patients. Hence, a thorough investigation of these factors is warranted nationwide.

## 5. Conclusion

More than forty percent of patients acquired target organ damage which is more significant. Heart failure is the most common form TOD in hypertensive patients. Factors such as presence of comorbidities, nonadherence to practice guidelines, and failing to change regimen were correlated with the incidence of target organ damage. Patient who receive medications according to standard treatment develop TOD very late as compared to those who took medication without the standard treatment guideline. Early diagnosis and management of hypertension and modification of triggering factors are essential to prevent complications. Moreover, drug prescription should be based on the standard treatment guidelines.

## Figures and Tables

**Table 1:** The sociodemographic and clinical characteristics of participants (*N* = 612).

Clinical characteristics	*N* (%)
Age (mean ± SD)	52.43 ± 13.2
Sex	
Females	341 (55.72)
Males	271 (44.28)
Residence	
Urban	459 (75)
Rural	153 (25)
Type of hypertension	
Systolic	160 (26.14)
Diastolic	50 (8.17)
Both	402 (65.69)
Duration of appointment	
One month	236 (38.56)
Two months	264 (43.14)
≥three months	112 (18.30)
Comorbidities	
Yes	222 (36.27)
No	390 (63.73)
Target organ damage (TOD)	
Yes	247 (40.36)
No	365 (59.64)

**Table 2:** The incidence rate of TOD in hypertensive patients' 1000/person/year. A person-time analysis.

Complication	Incidence/1000 person/year
CKD	9
Retinopathy	2.44
LVH	5.4
IHD	22
HF	30.8
Valvular heart disease	1.46
Stroke	14.65
Atrial fibrillation	2.44
Hypertensive urgencies	17.55
Sexual dysfunction	0.49

**Table 3:** The overall prevalence of TOD in hypertensive patients.

Type of complication	Prevalence *N* (%)
Retinopathy	6 (0.9)
LVH	12 (2)
IHD	51 (8.3)
HF	75 (12.2)
CKD	20 (3.3)
Hypertensive urgency	39 (6.4)
Sexual dysfunction	4 (0.67)
Stroke	34 (5.5)
AF	6 (0.9)
Total TOD	247 (100.0)

**Table 4:** Predictors of the target organ damage due to hypertension: multivariate cox regression.

Variables	Complication (yes)	Complication (no)	CHR (95%)	AHR (95% CI)
Sex (Male)	126 (20.59)	145 (23.69)	0.902 (0.693–1.174)	0.768 (0.563–1.145)
Residence (rural)	78 (12.75)	75 (12.25)	1.245 (0.923–1.679)	1.261 (0.824–1.759)
Presence of comorbidities	118 (19.3)	104 (16.99)	1.073 (1.01–1.437)	1.596 (1.174–1.637)^*∗*^
HTN (diastolic)	21 (3.43)	29 (4.74)	0.782 (0.57–1.087)	0.635 (0.448–1.536)
Both	179 (29.25)	223 (36.44)	0.713 (0.387–1.831)	0.526 (0.327–1.725)
Duration of appointment				
Two months	118 (19.28)	146 (23.6)	2.167 (0.295–5.19)	4.548 (0.591–6.423)
≥Three months	35 (5.72)	77 (12.58)	1.176 (0.776–1.781)	1.094 (0.709–1.689)
Controlled BP	199 (32.52)	282 (46.10)	0.605 (0.411–0.839)	0.771 (0.507–0.974)^*∗*^
Discordant with JNC	176 (28.76)	279 (45.59)	1.537 (1.167–2.024)^*∗*^	1.636 (1.189–2.251)^*∗*^
Absence of regimen change	191 (31.21)	312 (50.98)	1.702 (1.240–2.336)^*∗∗*^	1.857 (1.325–2.602)^*∗∗*^
Therapy				
Dual therapy	138 (22.55)	153 (25.0)	0.832 (0.642–1.080)	0.79 (0.473–1.786)
Triple therapy	56 (9.15)	53 (8.66)	0.715 (0.511–1.001)	1.317 (0.629–1.852)
Age				
31–40	32 (5.23)	41 (6.70)	0.75 (0.349–1.612)	0.732 (0.332–1.617)
41–50	44 (7.19)	53 (8.66)	0.995 (0.621–1.594)	0.975 (0.599–1.587)
51–60	60 (9.80)	98 (16.01)	0.89 (0.573–1.382)	0.745 (0.587–1.536)
61–70	64 (10.46)	82 (13.40)	0.797 (0.534–1.190)	0.862 (0.512–1.163)
≥70	47 (7.68)	53 (8.66)	0.904 (0.607–1.347)	0.836 (0.477–1.421)
Duration of hypertension				
Three years and below	19 (3.10)	41 (6.70)	1	1
Four years	14 (2.29)	25 (4.10)	1.216 [0.823–2.572]	1.211 [0.584–2.635]
Five years	70 (11.44)	108 (17.65)	1.276 [0.815–3.265]	1.355 [0.674–3.481]
Six years	105 (17.16)	136 (22.22)	1.241 [0.736–2.341]	1.316 [0.622–2.415]
Seven years	39 (6.37)	55 (8.99)	2.592 [2.35–6.332]	2.716 [1.632–7.082]^*∗*^
Class of hypertensive medications				
ACIES	15 (2.45)	27 (4.41)	1	1
BBCs	20 (3.27)	24 (3.92)	2.951 [2.651–5.535]	2.974 [2.631–7.072]^*∗*^
CCBs	17 (2.78)	21 (3.43)	0.614 [0.143–2.639]	0.517 [0.137–2.174]
Diuretics	14 (2.29)	25 (4.08)	1.654 [0.727–3.974]	1.521 [0.689–3.454]
Diuretics + ACEIs	70 (11.44)	80 (13.07)	1.375 [0.146–3.369]	1.528 [0.636–3.158]
Diuretics + *β*-blocker	68 (11.11)	73 (11.93)	1.146 [0.765–2.321]	1.256 [0.674–2.117]
B-blockers + ACEIs + diuretics	56 (9.15)	53 (8.66)	1.248 [0.436–2.773]	1.210 [0.481–2.576]
Other combinations	21 (3.43)	28 (4.57)	1.431 [0.726–2.372]	1.431 [0.783–2.541]

ACEIS: angiotensin convertase inhibitors, CCBs: calcium channel blockers, BBCs: beta-blockers, CHR: crude hazard ratio, and AHR: adjusted hazard ratio, ^*∗*^significant at 0.05 level. Female, urban residence, absence of comorbidity, systolic hypertension, one-month duration of appointment, uncontrolled BP, concordance with guidelines, presence of regimen change, monotherapy, and age between 21 and 30 were our reference variables. *∗∗* indicates significance at 0.01 level.

## Abbreviations

AOR: Adjusted odds ratio
BP: Blood pressure
COR: Crude odds ratio
GUH: Gondar university hospital
HF: Heart failure
HPs: Hypertensive patients
HTN: Hypertension
IHD: Ischemic heart disease
IR: Incident rate
JNC: Joint national committee
LVH: Left ventricular hypertrophy
SPSS: Statistical package for social sciences
TOD: Target organ damage
UBP: Uncontrolled blood pressure.

## Data Access

The datasets used during the current study are available from the corresponding author on reasonable request.
